# Diet-induced reconstruction of mucosal microbiota associated with alterations of epithelium lectin expression and regulation in the maintenance of rumen homeostasis

**DOI:** 10.1038/s41598-017-03478-2

**Published:** 2017-06-21

**Authors:** Hong Shen, Zhongyan Lu, Zhihui Xu, Zanming Shen

**Affiliations:** 1 College of Life Science, Nanjing Agricultural University, Nanjing, Jiangsu China; 2 Bioinformatics Center, Nanjing Agricultural University, Nanjing, Jiangsu China; 3 Key Lab of Animal Physiology and Biochemistry, College of Veterinary Medicine, Nanjing Agricultural University, Nanjing, Jiangsu China

## Abstract

It is unknown whether lectins of the rumen epithelium contribute to the recognition of mucosal microbes and activation of tolerogenic cytokines in ruminant animals. We applied an integrated method of RNA-seq and 16S rRNA gene sequencing to investigate alterations of epithelial lectin expression and regulation with a diet-induced reconstruction of the mucosal microbiota in the goat rumen. Our results showed that the diversity and richness of the rumen mucosal microbiota were promoted by the dietary concentrate. Meantime, in the rumen epithelium, five lectin genes, namely, sialic acid-binding Ig-like lectin 14 (LOC102180073), C-type lectin domain family 4, member E (CLEC4E), C-type lectin domain family 7, member A (CLEC7A), C-type lectin domain family 16, member A (CLEC16A), and lectin, mannose-binding 2 (LMAN2), were indicated to promote the expression of 8 tolerogenic cytokines, transforming growth factor beta 1 (TGFB1) and 4 enzyme genes involved in retinoic acid biosynthesis via 6 signaling pathways. Analysis of the combined data showed that 9 microbial genera (Clostridium_IV, *Desulfobulbus*, *Eubacterium*, *Ochrobactrum*, *Propionibacterium*, *Pseudomonas*, *Slackia*, *Staphylococcus* and Subdivision5_genera_IS) were highly related to the expression of functional lectins. These findings provide new insights into the interactions between the rumen epithelium and mucosal microbiota in the maintenance of rumen homeostasis.

## Introduction

The mucosal microbiota is a group of gastrointestinal (GI) microbiota that inhabits the mucus layer of the GI tract. Compared with the group inhabiting the fluid phase of the GI tract, the mucosal microbiota plays a more critical role in the development and performance of the immune functions of the GI epithelium, as well as the whole organism^1, 2^. Throughout evolution, the GI epithelium has developed a complex gene network to regulate the activities of the mucosal microbiota and responses of immune cells to provide a harmonious coexistence between the host’s immune system and GI microbiota. In healthy individuals, this network recognizes commensal bacteria in the mucus layer and subsequently activates tolerogenic signals to suppress inappropriate immune responses^3, 4^. Thus, it plays a crucial role in the maintenance of GI homeostasis. However, to date, it is unclear how the GI epithelium recognizes mucosal bacteria and which types of signals are involved in the communication between the GI epithelium and mucosal microbiota.

Lectins are a class of genes whose sequences have several carbohydrate-recognition domains (also referred to as lectin domains)^5^. They are evolutionally conserved pattern-recognition receptors (PRRs) that mediate the agglutination and immobilization of recognized pathogens^6^. Some lectins, such as mannose-binding lectin (MBL) and ficolin, are established as activators of the complement system, which plays important roles in the promotion of the innate and adaptive immunity responses^7, 8^. To date, more than 10 lectin families have been identified in eukaryotes according to sequence differences of the lectin domains^9^. Accumulating evidence has shown that members from three lectin families, C-type lectins, galectins and siglecs, play crucial roles in suppressing the immune response by recognizing O-glycan-coated cells (including bacteria) in mammals^10^. For example, mucin-induced activation of C-type lectins on human dendritic cells (DCs) promotes DC secretion of the tolerogenic cytokines interleukin (IL)-10 and transforming growth factor (TGF)-α^11^. Tumor cell-induced activation of siglecs on antigen-presenting cells (APCs) of mice leads to APC secretion of IL-10, TGFs and chemokines^12^. Subsequently, tolerogenic cytokines, as well as TGF and retinoic acid (RA), promote the generation of regulatory T cells (Tregs), which play important roles in the suppression of inappropriate immune responses in the GI epithelium^13^. Although there is currently no available report concerning the functions of lectins of the GI epithelium in the regulation of immune tolerance, lectins are present and constantly expressed in the GI epithelium of animals^14–16^. Accordingly, we infer that these lectins contribute to GI homeostasis by recognizing nonpathogenic bacteria that inhabit the mucus layer and elicit tolerogenic signals in the GI epithelium.

The rumen is the most important digestive and absorptive organ, as well as the most crucial habitat of symbiotic bacteria, in ruminant animals. Previous studies have shown that the dietary concentrate facilitates rumen fermentation and consequently affects the structure of the mucosal microbiota in the rumen^17^. We found that the dietary concentrate promoted immunity of the rumen epithelium by strengthening signaling from toll-like receptors (TLRs)^18^. However, it is unknown which members of the lectin families are expressed in the rumen epithelium and which regulatory mechanisms these lectins are used for rumen homeostasis. RNA-seq is a powerful technology for determining the expression and regulatory mechanisms of less-informed gene families. In this study, we shifted the dietary concentrate from 10% to 35% to induce reconstruction of the mucosal microbiota in the goat rumen. After that, we used 16S rRNA gene sequencing to investigate alterations of the mucosal microbiota and used RNA-seq to select the candidate lectins that are involved in the maintenance of rumen homeostasis. Finally, we used statistical tests to determine the regulatory pathways and tolerogenic signals related to the candidate lectins and identify the covariance of the mucosal bacteria and lectin transcripts. By this process, we aimed to understand the regulatory mechanism of lectins in the maintenance of rumen homeostasis.

## Results

### Histomorphometric Analysis of Rumen Epithelium

The 35% concentrate (MC) diet led to an increase in the ruminal papillae length, width, density, and number of cell layers compared with the 10% concentrate (LC) diet. However, in all samples, the morphology of the rumen epithelium remained complete.

### Structure of Epithelium Bacterial Communities

**Figure 1:**
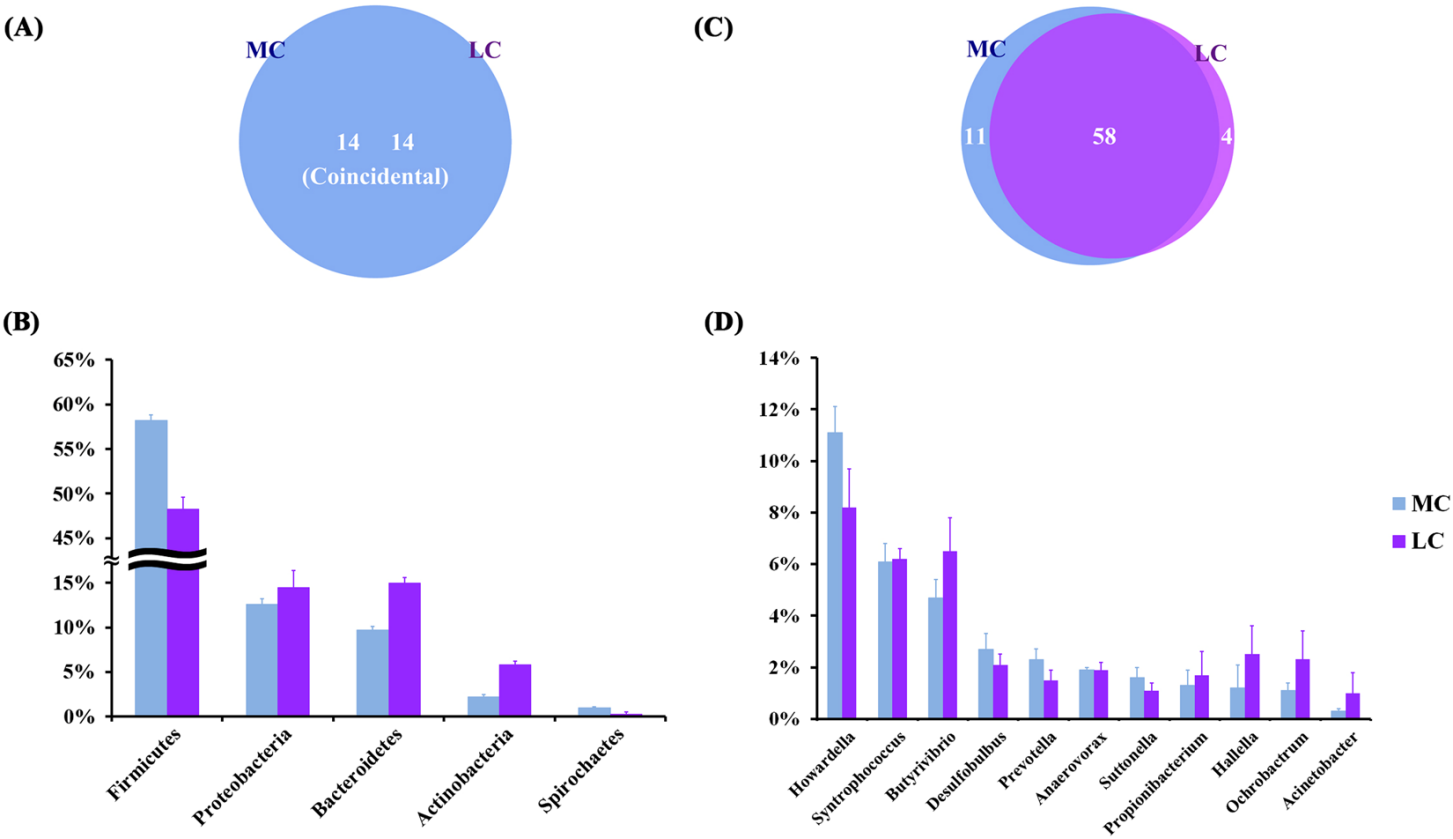
(**A**) Venn diagram showing the coincidence of phyla between the groups. (**B**) Phylum-level comparison of bacterial OTUs between the groups. (**C**) Venn diagram showing the overlap of genera between groups. (**D**) Genus-level comparison of bacterial OTUs between the groups.

At the phylum level, a total of 14 prokaryotic phyla were identified with 97% similarity, and all of them were common to both groups (Fig. 1A). Firmicutes (23.3–33.2%), Proteobacteria (12.6–14.5%), and Bacteroidetes (9.7–15.0%) were the most abundant among all microbial communities. Compared with the LC group, the relative abundance of Spirochetes was increased by 230%, Verrucomicrobia was increased by 50%, and Firmicutes was increased by 42% in the MC group. By contrast, the relative abundance of the remaining phyla was decreased. The most significant decrease occurred in the phylum Actinobacteria, whose relative abundance was decreased by 62% in the MC group. At the genus level, except for unclassified OTUs, a total of 73 genera were detected in the sequences. Among them, 58 genera were common to both groups (Fig. 1C). The abundances of all of the genera in both groups are shown in Table S2. *Howardella* (11.1–8.2%) was consistently abundant in both groups, except for the unclassified OTUs at the genus level. Four genera were only detected in the LC group, and 11 genera were only detected in the MC group. Nonmetric multidimensional scaling (NMDS) plots (Fig. S1) and analysis of similarities (ANOSIM) (*p* < 0.05) revealed the divergence of the community structure in the MC and LC groups.

### Diversity and Richness of Microbial Communities

**Figure 2:**
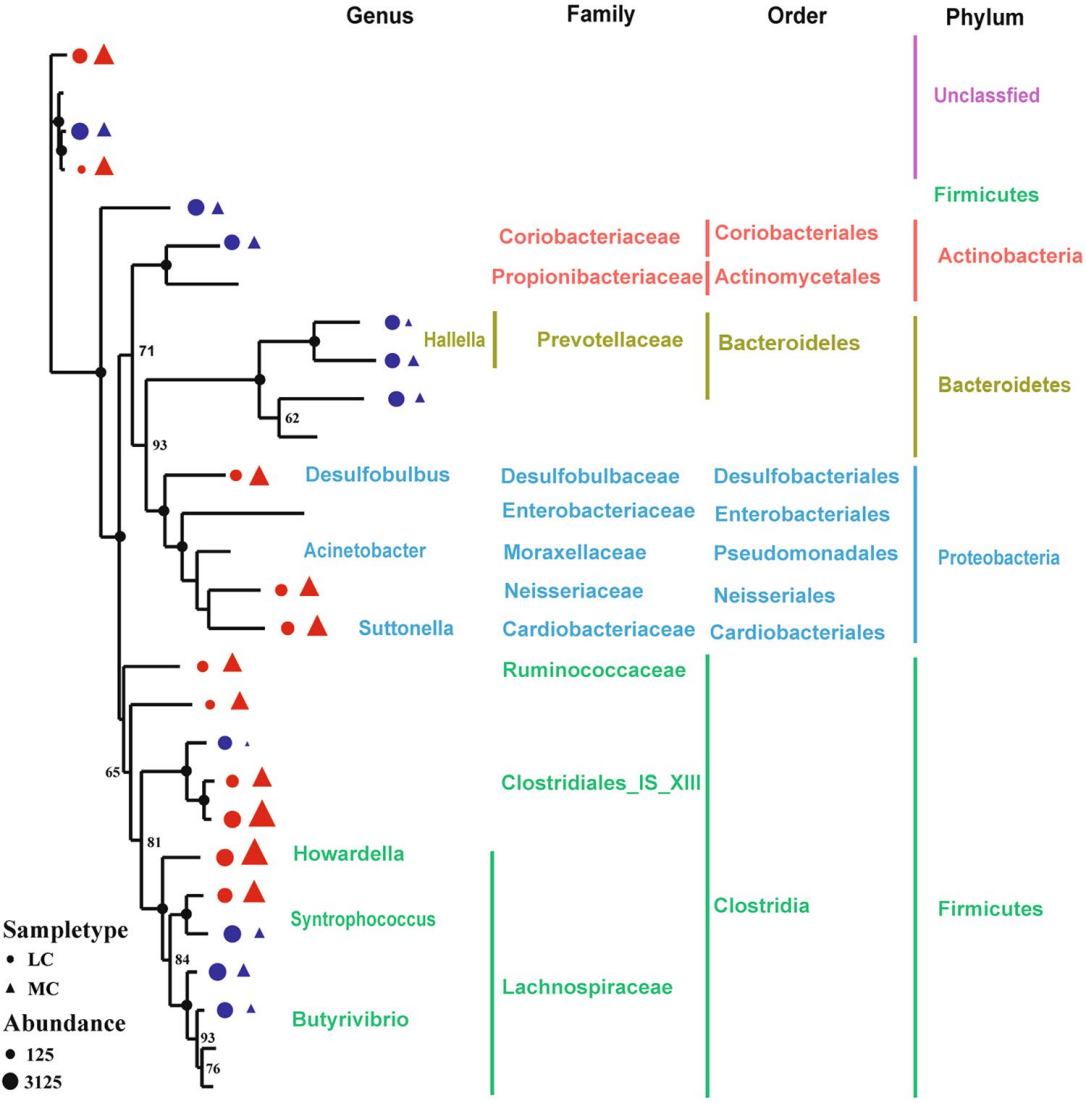
Maximum likelihood tree of 28 detectable OTUs (the relative abundance >1% in the given sample). The complete 16S rRNA gene sequences of the corresponding species in the RDP database were used to construct the tree. The triangle indicates the OTUs in the MC group, and the circle indicates the OTUs in the LC group. Only the OTUs with significantly different (*p* < 0.05) relative abundances are shown behind the branches. The size of the symbol indicates the relative abundance of the OTUs. Red indicates a significant expansion (*p* < 0.05) of the relative abundance of the OTU under the 35% concentrate diet and blue indicates a significant reduction (*p* < 0.05) in the relative abundance of the OTU under a 10% concentrate diet. Only those bootstrap values greater than 60 are shown on the tree. The solid black circles at the nodes stand for a bootstrap value of 100.

The diversity of the microbial community significantly decreased in the MC group, as indicated by the Shannon index. At the phylum level, the diversity of Bacteroidetes in the MC group was significantly higher than that in the LC group (*p* < 0.05). The diversity of Proteobacteria and Firmicutes in the MC group was significantly lower than that in the LC group (Fig. S2). Maximum likelihood (ML) analysis of 28 detectable OTUs (the relative abundance >1%) showed that the significantly expanded OTUs in the MC group belonged to the families Desulfobulbaceae, Neisseriaceae, Cardiobacteriaceae, Ruminococcaceae, and Clostridiales_IS_XIII (Fig. 2). By contrast, the significantly decreased OTUs belonged to the families Coriobacteriaceae and Prevotellaceae. OTUs belonging to the family Lachnospiraceae showed an inconsistent trend, with 2 OTUs expanding significantly and 3 OTUs shrinking significantly in the MC group.

A rarefaction curve (Fig. S3) was applied to evaluate the richness of the microbiota in each given sample. Compared with the MC group, these curves revealed that the OTU number was significantly increased in the LC group.

### Expression Profiles of Lectins in the Rumen Epithelium

The RNA-seq method was used to identify the repertoire of transcribed lectins in the rumen epithelium. It generated a total of 135 M raw reads (average 22.5 M reads per sample, range 20.9–23.5 M) and 127 M clean reads (average 21.2 M reads per sample, range 19.1–22.5 M). On average, 95% of the reads were successfully mapped to the NCBI goat genome annotation release version 101.

One hundred members of the lectin families were found to be encoded on the goat genome. The transcriptome data showed that 35 members were expressed in the rumen epithelium (reads per kilobase million; RPKM >1 in at least one sample) (Table 1). Compared with expression in the LC group, we found that 3 members were significantly upregulated (log_2_(MC/LC) >1) and 10 members were significantly downregulated (log_2_(MC/LC) <1) in the MC group. By comparing the expression among the members, we found that the expression of galectin-7 (LOC102180339) was highest in all samples (376 RPKM-513 RPKM). Meanwhile, six other members expressed more than 100 RPKM in at least one group.

**Table 1:** Members of the lectin families expressed and observed in the present study.

Item	Symbol	Description	MC (RPKM)	LC (RPKM)	Log2 (MC/LC)	Lectin Family
1	LOC102180073	sialic acid-binding Ig-like lectin 14	0.03	1.33	−5.47	siglec
2	FCN1	ficolin 1	0.93	4.98	−2.42	galectin
3	CLEC7A	C-type lectin domain family 7, member A	0.65	1.84	−1.50	CLR
4	LOC102181776	sialic acid-binding Ig-like lectin 14	0.78	1.89	−1.28	siglec
5	LOC102184901	C-type lectin domain family 2 member D11-like	65.59	147.84	−1.17	CLR
6	CLEC4E	C-type lectin domain family 4, member E	1.27	2.82	−1.15	CLR
7	LGALS9	lectin, galactoside-binding, soluble, 9	5.40	11.44	−1.08	galectin
8	CLEC16A	C-type lectin domain family 16, member A	2.11	4.35	−1.04	CLR
9	LGALS3BP	lectin, galactoside-binding, soluble, 3 binding protein	3.24	6.65	−1.04	galectin
10	LMAN2	lectin, mannose-binding 2	30.76	62.86	−1.03	CLR
11	LGALS15	lectin, galactoside-binding, soluble, 15	1.50	0.64	1.23	galectin
12	LOC106503212	C-type lectin domain family 10 member A-like	3.99	1.96	1.03	CLR
13	LOC102189655	C-type lectin domain family 2 member D11-like	2.48	1.24	1.00	CLR
14	LOC102180339	galectin-7	393.68	486.38	−0.31	galectin
15	LOC102180072	galectin-7	280.25	327.80	−0.23	galectin
16	CLEC3B	C-type lectin domain family 3, member B	263.21	205.23	0.36	CLR
17	LGALS3	lectin, galactoside-binding, soluble, 3	99.80	108.23	−0.12	galectin
18	LGALSL	lectin, galactoside-binding-like	114.98	99.52	0.21	galectin
19	LGALS1	lectin, galactoside-binding, soluble, 1	148.30	86.79	0.77	galectin
20	OS9	OS9, endoplasmic reticulum lectin	42.99	49.58	−0.21	CLR
21	ERLEC1	endoplasmic reticulum lectin 1	25.02	21.84	0.20	CLR
22	LOC106504001	C-type lectin domain family 1 member A-like	20.86	20.86	0.00	CLR
23	LMAN1	lectin, mannose-binding, 1	12.35	14.85	0.00	CLR
24	LGALS8	lectin, galactoside-binding, soluble, 8	14.36	14.59	0.27	galectin
25	LGALS4	lectin, galactoside-binding, soluble, 4	10.33	7.80	0.02	galectin
26	LOC102180574	C-type lectin domain family 6 member A	7.15	6.63	−0.41	CLR
27	COLEC12	collectin sub-family member 12	6.82	5.96	−0.11	CLR
28	LOC102180291	C-type lectin domain family 4 member A	4.92	3.52	−0.19	CLR
29	CLEC5A	C-type lectin domain family 5, member A	1.63	2.01	−0.48	CLR
30	CLEC12A	C-type lectin domain family 12, member A	2.17	1.82	0.30	CLR
31	CLEC2A	C-type lectin domain family 2, member A	2.89	1.69	−0.25	CLR
32	CLEC1A	C-type lectin domain family 1, member A	2.47	1.51	−0.77	CLR
33	LMAN2L	lectin, mannose-binding 2-like	0.89	1.50	−0.71	CLR
34	CLEC14A	C-type lectin domain family 14, member A	0.88	1.36	0.75	CLR
35	LOC102189932	C-type lectin domain family 2 member H-like	1.66	1.02	0.63	CLR

### Related KEGG Pathways of Differentially Expressed Lectins

**Figure 3:**
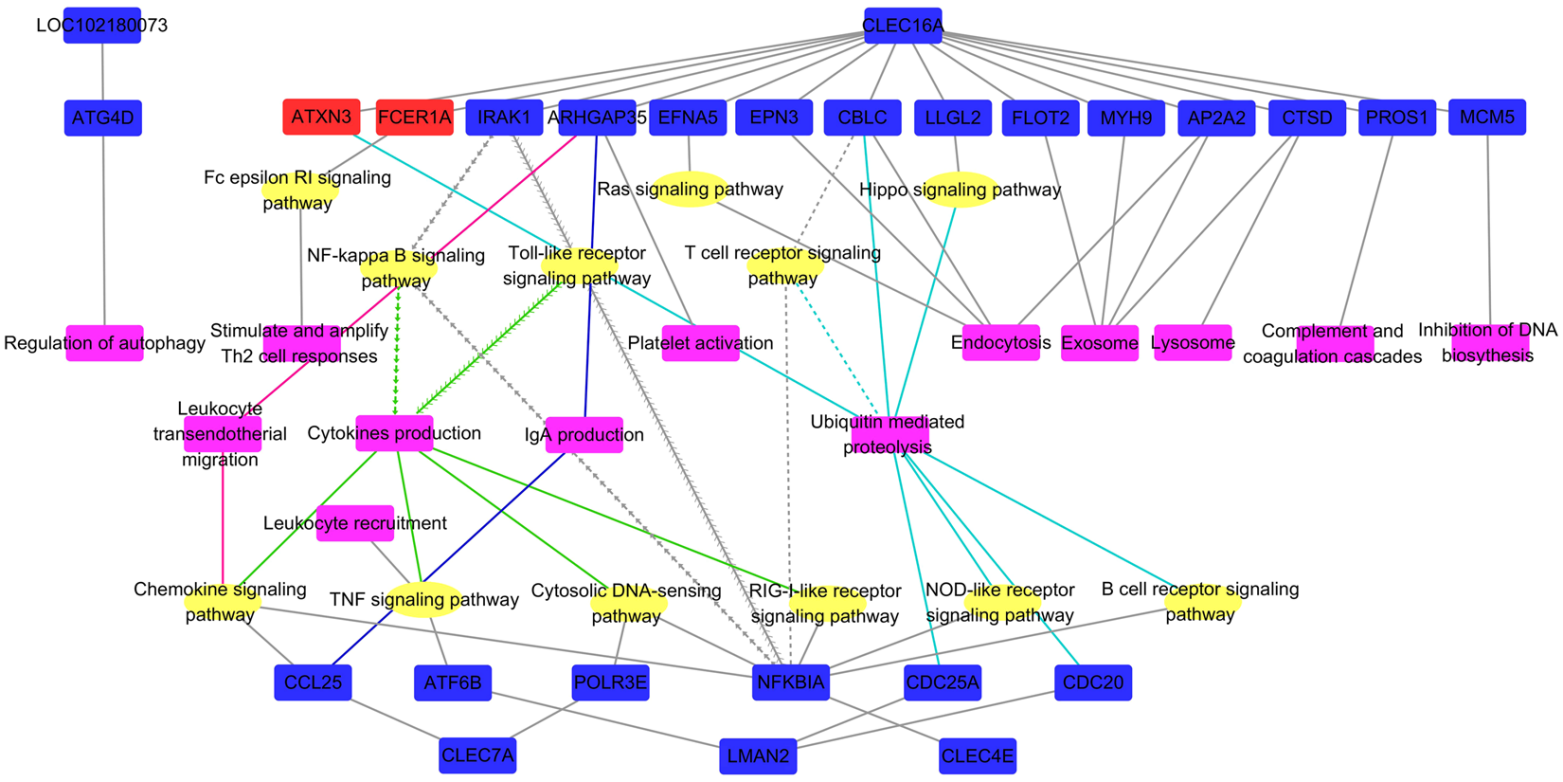
Regulation network of the lectin genes related to immune activities. The functions of the first neighbors were predicted by KEGG pathway analysis. Genes that show positive correlation with the 35% concentrate feeding are in red, and the genes that show negative correlation with the 35% concentrate diet are in blue. Signaling pathways are in yellow, and regulatory functions are in violet. The genes regulating the same signaling pathways are given the same line shapes and the genes and signaling pathways regulating the same functions are given the same color.

We used the spearman correlation coefficient (SCC) to quantify gene–gene co-expression for all of the gene-pairs in the transcriptome across all samples. To assess the biological significance of the lectin co-expression network, we checked the related signaling pathways and possible functions of the first neighbors by the Kyoto Encyclopedia of Genes and Genomes (KEGG) annotation. The functions of the significantly different neighbors were classified according to the regulated physiological processes of the rumen epithelium. Accordingly, the regulatory pathways involved in the modulation of immune activities and cytokine productions in this study were assembled and referred to as the immune regulation network (Fig. 3). Other possible types of regulation involved in the cellular community, development, metabolism, and transcription modulation are listed in Table S3.

Five members of the lectin families that had upregulated expression in the LC group were involved in the regulation of immune activities in the rumen epithelium: sialic acid-binding Ig-like lectin 14 (LOC102180073), C-type lectin domain family 16, member A (CLEC16A), C-type lectin domain family 7, member A (CLEC7A), mannose-binding lectin 2 (LMAN2L), and C-type lectin domain family 4, member E (CLEC4E). Except for the negative correlations between the expression of CLEC16A and ataxin 3 (ATXN3) as well as the Fc fragment of immunoglobulin E receptor 1a (FCER1A), there were positive correlations for all gene pairs. Endocytosis, exosome, lysosome, complement and coagulation, inhibition of DNA biosynthesis, immunoglobulin A (IgA) production, platelet activation, and autophagy were directly regulated by neighboring genes. However, leukocyte trans-endothelial migration, leukocyte recruitment, stimulation and amplification of Th2 cell responses, and cytokine production were regulated via activation of specific relevant signaling pathways. The ubiquitin system was involved in lectin modulation of proteolysis.

### Differentially Expressed Cytokines

Comparing the expression of the genes encoding ILs, TGFs and chemokines between groups, we found that the expression of 8 cytokine genes and TGFB1 was significantly upregulated in the LC group (Table 2). Among the differentially expressed ILs, IL22 was reported to be secreted by the group 3 innate lymphoid cells in the mucosal tissue and to play important roles in the maintenance of immune homeostasis^19^. IL19, belonging to the IL-10 family, was reported to be an anti-inflammatory cytokine^20^. Other mentioned genes and their functions are listed in Table 2 ^21–25^.

**Table 2:** Differentially expressed genes encoding interleukins, transforming growth factors and chemokines.

Symbol	Description	MC (RPKM)	LC (RPKM)	Log2 (MC/LC)	Reported Function in GI epithelium
IL22	interleukin 22	2.11	11.27	−2.42	Group 3 ILC derived homeostatic cytokine^16^
IL19	interleukin 19	84.30	306.20	−1.86	IL-10 family anti-inflammatory cytokine^17^
IL1B	interleukin 1 beta	6.95	14.43	−1.05	Pro-inflammatory cytokine
IL26	interleukin 26	7.69	3.34	1.20	Regulation of intercellular adhesion^21^
IL33	interleukin 33	24.37	11.53	1.08	Regulation of cell cycle^20^
LOC102170310	C-C motif chemokine 15	12.80	29.18	−1.19	Recruitment of monocytes, T cells, eosinophils^19^
LOC102181154	C-C motif chemokine 3	1.21	3.35	−1.47	Recruitment of monocytes, T cells, NK cells, basophils, eosinophils^19^
CXCL8	C-X-C motif chemokine ligand 8	41.09	153.22	−1.90	Recruitment of neutrophils, T cells, basophils, endothelial cells^19^
CX3CL1	C-X3-C motif chemokine ligand 1	0.80	2.35	−1.56	Recruitment of effector T cells^19^
CXCL11	C-X-C motif chemokine ligand 11	2.06	5.08	−1.30	Recruitment of T cells^19^
LOC102174969	C-C motif chemokine 8	11.57	5.16	1.17	Recruitment of monocytes, T cells, eosinophils, basophils, NK cells^19^
CXCL14	C-X-C motif chemokine ligand 14	194.03	86.38	1.17	Recruitment of neutrophils, NK cells^19^
TGFB1	transforming growth factor beta 1	1.39	3.54	−1.35	Regulation of DCs’ conversion^5^
RDH13	retinol dehydrogenase 13	2.86	6.31	−1.14	Metabolize retinol to retinaldehyde^18^
ALDH1A3	aldehyde dehydrogenase 1 family member A3	11.92	25.31	−1.09	Metabolize retinaldehyde to retinoic acid^18^
ALDH16A1	aldehyde dehydrogenase 16 family member A1	2.33	4.73	−1.02	Metabolize retinaldehyde to retinoic acid^18^
ALDH4A1	aldehyde dehydrogenase 4 family member A1	2.90	8.32	−1.52	Metabolize retinaldehyde to retinoic acid^18^
ALDH1A1	aldehyde dehydrogenase 1 family member A1	1420.73	558.50	1.35	Metabolize retinaldehyde to retinoic acid^18^

### Relationships between Lectin Expression and the Relative Abundance of Bacterial Genera

**Figure 4:**
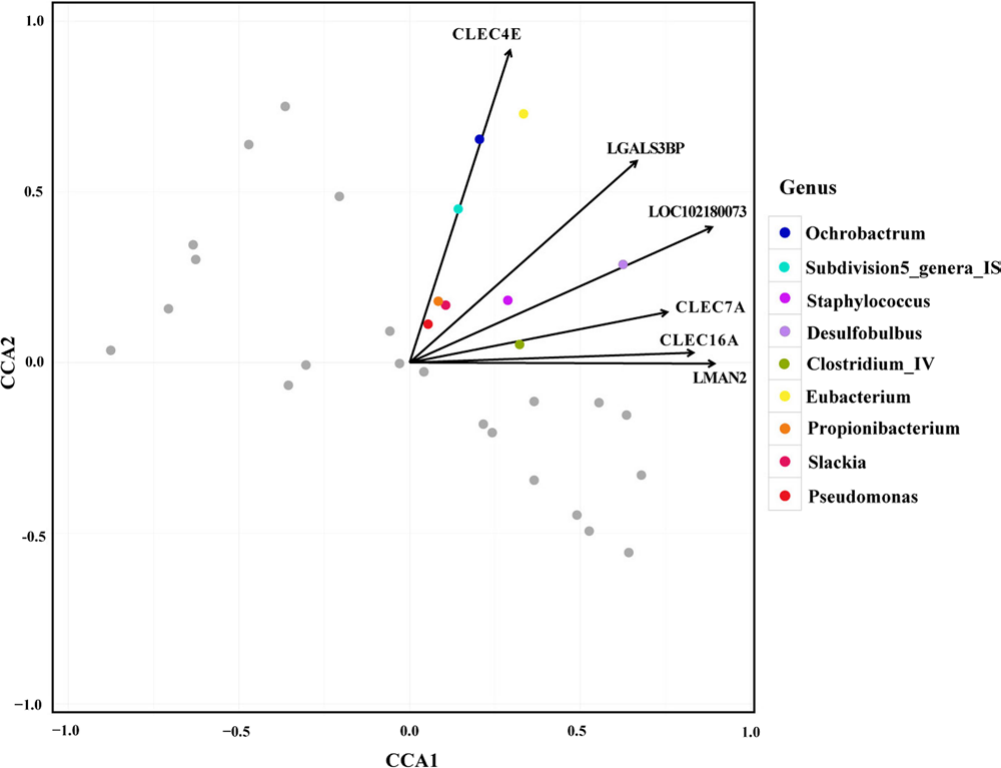
Constrained correspondence analysis revealing the correlations between the relative abundance of the reduced microbial clades and the expression of the functional lectin genes.

Spearman correlation analysis removed 90% of the OTUs (585/647), which have a SCC with differentially expressed lectins of the correlation coefficient less than 0.6 and *p*-value greater than 0.05. Subsequently, constrained correspondence analysis (CCA) showed positive correlations between functioning lectins (referred to as the lectins that take part in the above-mentioned regulation networks) in 9 expanded microbial genera. The microbial genera that were related to functional lectin transcripts are listed in Fig. 4.

## Discussion

The GI tract is covered by a layer of heavily glycosylated proteins, commonly known as mucins. They are an important nutrient source for the microbiota that resides in the mucus layer. Previous studies have shown that the carbohydrate availability in the mucus layer affects the composition of the microbiota found there^26–28^. In the present study, an LC diet promoted diversification and expansion of the mucosal microbiota, indicating that the amount of energy substrates in the rumen fluid also has an impact on the composition of the mucosal microbiota. To date, the effects of dietary concentrations on the rumen mucosal microbiota are controversial. Mao *et al*.^29^ reported that a 65% dietary concentrate diet decreased the diversity of the mucosal microbiota in the goat rumen compared with a 0% concentrate diet. However, Wetzels *et al*.^30^ observed a positive relationship between dietary energy substrates and the diversity and richness of the mucosal microbiota in the goat rumen by using 0%, 30%, and 60% concentrate diets. The damage to the rumen epithelium after weeks of a zero or high concentrate diet might be the reason for these inconsistent results. Based on such considerations, we confirmed the integrity of the ruminal epithelium in all samples by using optical microscopy. Previous studies have shown that microbes with the ability to metabolize mucins have an advantage in colonization and expansion in the mucus layer. Except for unclassified OTUs, the detectable OTUs promoted by the LC diet in this study belong to the families Lachnospiraceae and Prevotellaceae. Members of Lachnospiraceae are reported to have the ability to degrade sialic acids and fucose in the mucus layer^31, 32^. Members of Prevotellaceae can degrade the core structure of the terminal mucin O-glycan^33^. Accordingly, our results support the idea that microbes that have the ability to metabolize carbohydrates in the mucosal layer have a competitive advantage in niches under a low energy diet.

Previous studies have indicated that separating microbes from the GI epithelium is a major task of the GI immune system in the maintenance of GI homeostasis^34^. According to the study by Vaishnava *et al*.^35^, the soluble lectins in the mucus layer play important roles in the spatial segregation of the microbiota and GI epithelium. Galectins, which include 15 known mammalian members, are soluble lectins located in the extracellular milieu^36^. In the present study, expression of galectin-7 was consistently highest in all samples, thereby indicating the constitutive roles of this lectin in the maintenance of rumen homeostasis. However, its expression was not synchronized with the expansion of the mucosal microbiota, suggesting a loose relationship between galectin-7 and the activities of the microbes.

To date, most C-type lectins (CLRs) and all known sialic acid-binding immunoglobulin-like lectins (siglecs) have been shown to be membrane-bound proteins that are found on the cell surface and that contain one or more carbohydrate-recognition domains (CRDs) that are responsible for glycan binding^10^. In the present study, one siglec gene (LOC102180073) and four CLR genes (CLEC4E, CLEC7A, CLEC16A, and LMAN2) were shown to be involved in 13 types of immune activities. Moreover, their expression was significantly increased with the expansion of the mucosal microbiota under the LC diet. Previous studies have shown that upon recognition of specific pathogens, innate immune cell CLRs contribute to DC differentiation and immune response programs by affecting cytokine production^37–41^. A well-described example is the signaling function of the activated CLR dectin-1 in the recognition of fungal α-glucan structures. Activated dectin-1 of DCs triggers expression of TNF-α, IL-10, and IL-12 by activating the p38-Erk-JNK kinase cascade and transcription factor NF-κB^40^. The CLR DC- specific intercellular adhesion molecule-3-grabbing nonintegrin (SIGN) of DCs has also been shown to signal upon recognition of pathogen glycan components, which trigger expression of IL-10 via TLR-dependent NF-κB signaling pathway^37^. In the present study, we observed that activation of CLRs had an impact on cytokine production by affecting 6 signaling pathways. In addition to the NF-κB and TLR signaling pathways, the chemokine signaling pathway, TNF signaling pathway, cytosolic DNA-sensing pathway, and RIG-I-like receptor signaling pathway were also involved in cytokine activation under the LC diet. Moreover, the co-expression network constructed in this study predicted that 4 additional mechanisms were involved in the programming functions of CLRs on the innate and adaptive immune responses, namely, leukocyte recruitment, leukocyte trans-endothelial migration, stimulation and amplification of Th2 cell responses, and platelet activation. Except for the inhibitory effects on the stimulation and amplification of Th2 cell responses, other activities appear to be enhanced by the LC diet. These activities, which are associated with an expanded microbiota, seem to create an alert environment for the immune system, with proper suppression of the inflammatory responses at the barrier site.

Alteration of microbial metabolic products in the mucus layer is a crucial reason for the change in lectin expression in the ruminal epithelium. However, since all known bacteria are coated with glycans, microbial composition alterations might also be a reason for the change in lectin expression. Over 20 pathogens are known to be recognized by lectins in animals. The ability to synthesize or capture glycans from their hosts and incorporate them into their own glycoconjugates is considered to be an important strategy to enable pathogens to escape an immune attack from the host’s immune system^10^. For example, an interaction between the high-mannose structures on the pathogen *Mycobacterium tuberculosis* and the CLR DC-SIGN on DCs leads to the production of anti-inflammatory cytokines, such as IL-10^42^. The glycan on the pathogen *Helicobacter pylori* activates the DC-SIGN on DCs, thereby inducing the conversion of Th1 into Th2^43^. In the present study, we predicted microbes that correlated with the expression of functioning lectins by statistical methods. Among them, members of *Desulfobulbus* are important mucosa-associated sulfate-reducing bacteria (SRB) in the human colon. They are thought to play roles in the pathogenesis of chronic inflammatory disorders of the colon^44^. Bacteria belonging to the lineage of the subdivision5_genera_IS have important functions in the suppression of immune responses and maintenance of GI homeostasis^45–47^. *Staphylococcus*, *Propionibacterium* and *Ochrobactrum* are potential pathogens to animals^48–50^. Members of *Eubacterium* and Clostridium_IV have been revealed to be major butyrate-producing bacteria in the human colon^51^. Members of *Slackia* have been shown to exhibit a high adhesion capacity to human Caco-2 cells^52^ and to also be involved in the pathogenesis of prostate cancer^53^. However, little information is presently available on the cell-wall components and metabolites of the gut bacteria that belong to these clades. Other than correlation analysis, we have not been able make further inferences regarding the interactions between the microbes and functioned lectins studied here. Further studies are required to identify their functions and significance in the maintenance of GI homeostasis.

In summary, we found that the low concentrate diet promoted colonization of opportunistic pathogens in the mucus layer of the rumen. Such alterations in the compositions of the mucosal microbiota induced upregulated expression of five membrane-bound lectins on the rumen epithelium. Subsequently, these five functional lectins contributed to the downregulated expression of 9 tolerogenic cytokines, TGFB1 and 4 enzyme genes involved in retinoic acid biosynthesis by downregulating the expression of genes related to six signaling pathways. Besides, the decrease of 9 microbial clades in the mucus layer was indicated to be correlated to upregulated expression of these functional lectins in the rumen epithelium. In sum, our study indicates that the lectins of the rumen epithelium play important roles in the transduction of microbial signals from the mucus layer to the immune system. They recognize specific bacteria in the mucus layer and, subsequently, induce tolerogenic signals in the rumen epithelium to suppress the attack of immune cells on nonpathogenic bacteria in the mucus layer. This study expands our fundamental knowledge concerning the roles of evolutionarily conserved lectins in the maintenance of rumen homeostasis and provides new insight into the interactions between the mucosal microbiota and rumen epithelium.

## Methods

### Ethics approval

This study was approved by the Animal Care and Use Committee of Nanjing Agricultural University, in compliance with the Regulations for the Administration of Affairs Concerning Experimental Animals (The State Science and Technology Commission of P. R. China, 1988) and the Care and Use of Animals (Nanjing Agricultural University, 1999).

### Animals

Six male goats (Boer × Yangtze River Delta White, aged 4 months) were randomly allocated into two groups and received either a diet of 65% hay plus 35% concentrate (MC group, *n* = 3) or a diet of 90% hay plus 10% concentrate (LC group, *n* = 3) (Table S1). All goats were fed with two equal portions of the designated diet at 0800 and 1700 daily for 28 days. Water was freely available to all goats during the experimental period. On day 28, the goats were killed at a local slaughterhouse.

### Sample Collection

On day 28, all goats were slaughtered at 8 h after the morning feeding. Rumen tissue from the ventral blind sac was quickly excised and gently washed using ice-cold phosphate-buffered saline (PBS; Ph 7.4). The epithelium was subsequently separated from the muscle layers and cut into 1–2 cm^2^ pieces. One piece was placed on ice and promptly used for the extraction of microbial DNA. Five pieces were immediately fixed in 4% paraformaldehyde (PFA) (Sigma, St. Louis, MO) for histomorphometric microscopy analysis. The remaining pieces were stored at −80 °C for later extraction of epithelial RNA.

### Morphological Study

Ten papillae per animal were prepared for optical microscopy analyzed according to the description of Odongo *et al*.^54^. In brief, single PFA-fixed papilla were embedded in paraffin and sectioned to a thickness of 6 µm. Each section was stained with hematoxylin and eosin and then mounted on a slide for microscopic analysis. Image Pro Plus software (Media Cybernetics, Silver Spring, MD) was used to observe the integrity of the epithelium in all samples.

### Microbial DNA Extraction and Sequencing

To detach the tightly attached bacteria, the ruminal epithelium was placed in a 1.5 ml tubes with 0.7 ml of PBS and several plastic beads and moderately shaken on a vortex for 30 second. The ruminal epithelium was moved to a new tube and processed with the detaching step once again. Subsequently, the metagenomic DNA was extracted from the PBS mixture by using a Bacterial DNA Kit (Omega, Shanghai, China). The DNA concentration was determined using a Nanodrop 1000 (Thermo Fisher Scientific, Wilmington, DE, USA) and stored at −20 °C until further processing. The 16S rRNA gene library preparation was performed by using polymerase chain reaction (PCR) amplification of the V3–V4 region. The universal primers 338F (5′-ACTCCTACGGGAGGCAGCAG-3′) and 806R (5′-GGACTACHVGGGTWTCTAAT-3′)^55^, which included TruSeq adapter sequences and indices, were used in the PCRs. All libraries were sequenced using an Illumina MiSeq platform (Illumina, San Diego, California, USA) at Biomarker Technologies, Beijing, China.

### Ruminal Microbiota Analysis

Paired reads were filtered for quality (Q30) and joined by FLASH v1.2.11^56^. Sequences that contained read lengths shorter than 400 bp and greater than 550 bp were removed by PRINSEQ v0.20.4^57^. The remaining sequences were then classified into operational taxonomic units (OTUs) by the RDP classifier release 9.0^58^ at the default setting. OTUs, whose counts were more than 3 in at least one of the samples, were hierarchically summed at all taxonomic levels, and the counts were normalized to the relative abundance for each sample. The diversity of the microbial communities was estimated using the R program phyloseq package^59^. For a deeper analysis of the diversity of the major evolutional clades in the mucosal microbiota, OTUs were filtered to a relative abundance of at least 1% in at least one sample. Then, MUSCLE v3.8.31^60^ was used to align the complete 16S rRNA sequences of the corresponding species in the RDP database. RaxML v8^61^ and the GTR model were used to construct the maximum likelihood (ML) trees. The R program ape package^62^ was used to plot the tree.

### Epithelial RNA Extraction and Sequencing

Total RNA was extracted from the ruminal epithelium using the RNAeasy Mini Kit (Qiagen, Shanghai, China) according to the manufacturer’s instructions. RNA was quantified using a NanoDrop 1000 spectrophotometer and its integrity was evaluated by using the RNA 6000 Assay Kit of the Agilent Bioanalyzer 2100 system (Agilent Technologies, CA, USA). High-quality RNA (RNA Integrity number >9.0) was processed using the NEBNext Ultra RNA Library Prep Kit (New England Biolabs, Beijing, China) following the manufacturer’s instruction. All libraries were sequenced via paired-end chemistry (PE125) on an Illumina HiSeq2500 platform (Illumina, San Diego, California, USA) at Biomarker Technologies, Beijing, China.

### Transcriptome Assembling and Differentially Expressed Gene Identification

Low-quality reads (including more than 50% low-quality bases (<Q30) and more than 10% ambiguous bases (N)) were first removed by using PRINSEQ v0.20.4. The NCBI goat genome annotation release version 101 was used to construct the reference genome by using Bowtie release 1.2.0^63^. High-quality reads were mapped to the reference genome using TopHat v2.1.0^64^ with standard parameters. Each SAM output file from the TopHat alignment was used in the Cuffdiff program of Cufflinks version 2.2.1^65^ as input files to test for differential gene expression. In the Cuffdiff program, only mapped reads were used to estimate the gene expression level of each gene transcript and the gene expression values were subsequently normalized to the RPKM. In this study, only genes with more than 1 RPKM in at least one of the samples were considered expressed.

### Gene Co-expression Network

The co-expressed genes were identified by computing the SCC between pairs of genes across six samples by using R program. Only expressed genes (RPKM > 1 in at least one sample) were used in the correlation analysis. A threshold for the SCC larger than 0.8 and *p*-value less than 0.05 was used to identify significantly co-expressed genes. The expression levels of the neighbors nearest to the differentially expressed lectins on the co-expression network were compared between the groups. The differentially expressed neighbors were analyzed from the pathways in the KEGG on the KABAS version 3.0 web server^66^. Finally, cytoscape version 3.4.0^67^ was applied to visualize the gene co-regulation network.

### Dimensionality Reduction for Microbial Features and Multi Data Integration

To improve the power to associate microbial composition with the transcriptional activity of the host genes, we reduced the dimensionality of the microbial features by calculating their SCC with the differentially expressed lectins. The procedure was the same as that used in the construction of the gene co-expression network. The OTUs, whose SCC were larger than 0.6 and *p*-value was less than 0.05 with at least one type of investigated lectins, were used in the following analysis. Next, the relationships between the relative abundance of the selected OTUs and the expressed RPKM of the differentially expressed lectins were explored using the CCA in the R program vegan package^68^. The expressed RPKM of the lectins was used as the environmental factors in the CCA analysis. The R program ggplot2 package^69^ was used to generate the visual interpretation of the gene-microbiota relationships. The location of each microbial genus was calculated as the centroid of the corresponding OTUs within the given genus.

### Availability of data and material

The meta-genome and transcriptome data are available in the NCBI under BioProject PRJNA339481.

## Electronic supplementary material

Supplementary Info File
