# Supplementary material for: Diet-induced reconstruction of mucosal microbiota associated with alterations of epithelium lectin expression and regulation in the maintenance of rumen homeostasis
Source: Sci Rep. 2017 Jun 21;7:3941. doi: 10.1038/s41598-017-03478-2 (PMC5479827; doi:10.1038/s41598-017-03478-2)
Supplement: Supplementary file 1 — Supplementary Info File [file 41598_2017_3478_MOESM1_ESM.docx]

# Diet-induced reconstruction of mucosal microbiota associated with alterations of epithelium lectin expression and regulation in the maintenance of rumen homeostasis

Hong Shen1,2, Zhihui Xu1.2, Zanming Shen3 and Zhongyan Lu3*

Fig. S1. Nonmetric multidimensional scaling (NMDS) analysis of Bray-Curtis similarity coefficients based on the relative abundance of OTUs in the given sample.


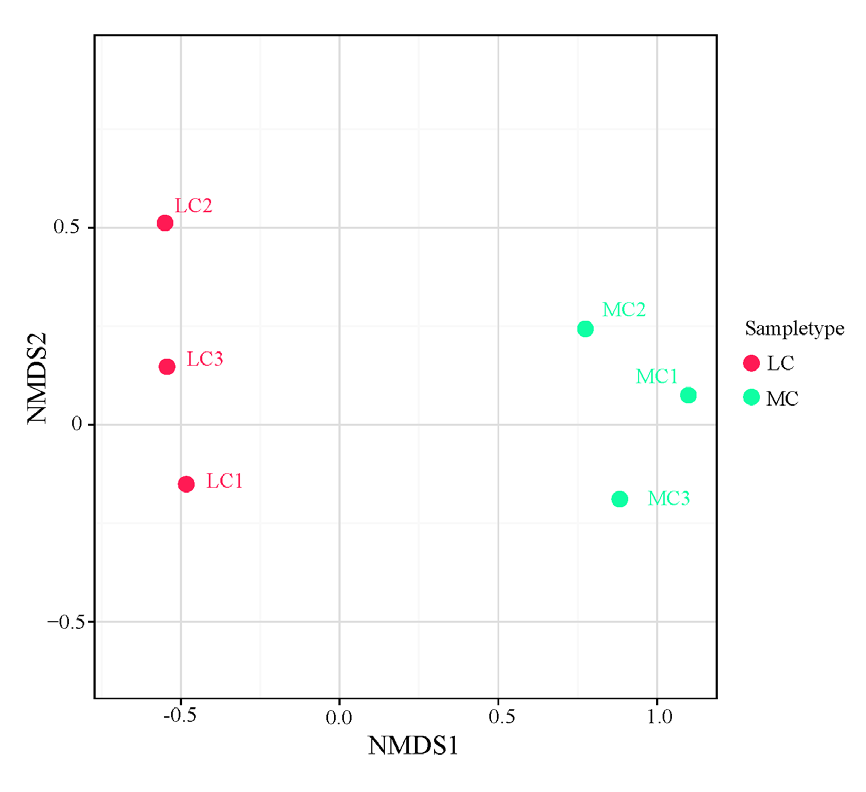


Fig. S2. (A) Diversity of the prokaryotic phyla estimated by using the Shannon index. (B) Diversity of the prokaryotic families estimated by using the Shannon index. (C) Diversity of the prokaryotic genera estimated by using the Shannon index.


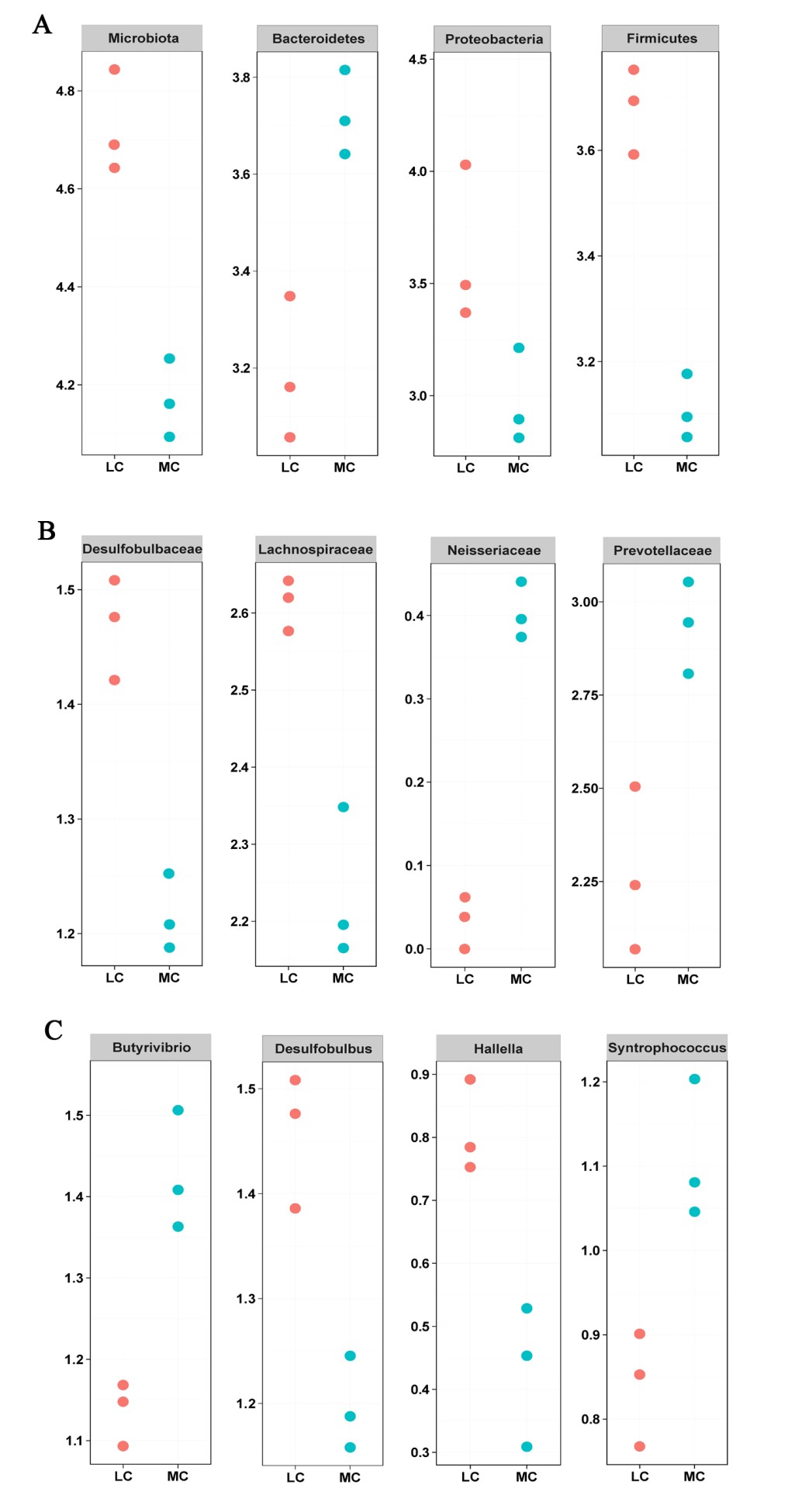


Fig. S3. Richness of the mucosal microbiota estimated by using rarefaction curves


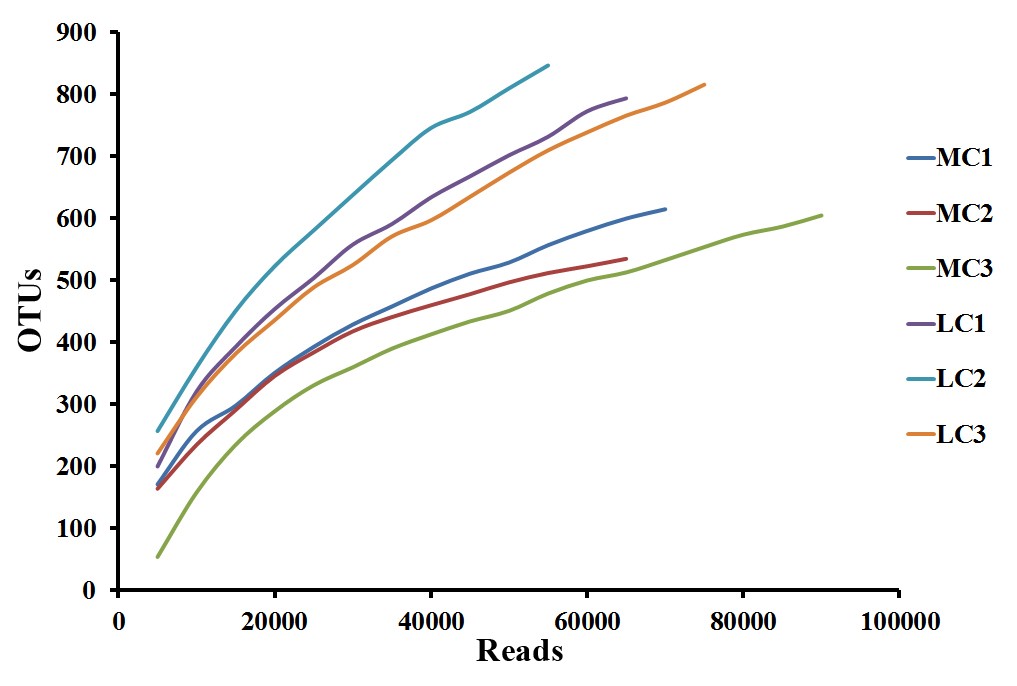


Table S1. Dry matter intake and chemical composition of diets in this study1

| Item | MC 2 | LC 2 |
| --- | --- | --- |
| **Dietary intake** | guinea grass + corn | guinea grass |
| Total DMI, g/d | 485.3 ± 6.4 | 432.4 ± 9.9 |
| NDF, g/d | 231.4 ± 4.5 | 289.0 ± 7.0 |
| ADF, g/d | 134.9 ± 2.7 | 172.9 ± 4.2 |
| NFC3, g/d | 165.0 ± 0.9 | 66.2 ± 1.4 |
| Initial body weight, kg ME, kJ/(kg0.75·d)  N, mg/(kg0.75·d) | 16.8±1.6  591.9 ± 7.8  1061.8 ± 14.0 | 16.7±1.0  500.7 ± 10.6  1014.0 ± 23.2 |
| **Ingredient, % of DM**  Guinea Grass | 65 | 90 |
| Corn | 25 | 0 |
| Soya bean meal | 8 | 8 |
| Additive4 | 2 | 2 |
| **Chemical composition** |  |  |
| DM,% | 89.9 | 91.4 |
| Crude protein,%DM | 10.7 | 10.3 |
| Crude fat, %DM | 3.4 | 3.5 |
| Crude fibre, %DM | 20.2 | 27.5 |
| Crude ash, %DM | 3.8 | 4.7 |
| NDF, %DM | 50.7 | 66.8 |
| ADF, %DM | 29.7 | 40.1 |
| NFC, %DM | 31.3 | 14.7 |
| ME,MJ/kg DM | 10.1 | 8.9 |

1 The values are means ± SE.

2 MC (n = 3), LC (n = 3)

3 NFC = 100 – (NDF + CP + crude fat + ash)

4 The additive was composed of calcium phosphate, limestone, trace mineral salt, and vitamin premix (vitamins A, D, and E).

**Table S2. 35% feeding resulted in a profound change in rumen microbial composition.**

| Item | Genus | MC | LC | MSE |
| --- | --- | --- | --- | --- |
| 1 | Acetivibrio | 50 | 59 | 43 |
| 2 | Acinetobacter | 281 | 1030 | 448 |
| 3 | Actinomycetospora | 14 | 0 | 7 |
| 4 | Alloprevotella | 135 | 55 | 85 |
| 5 | Anaerofustis | 44 | 0 | 22 |
| 6 | Anaeroplasma | 70 | 67 | 47 |
| 7 | Anaerorhabdus | 0 | 66 | 25 |
| 8 | Anaerovorax | 1889 | 1918 | 162 |
| 9 | Armatimonadetes_gp5 | 54 | 17 | 36 |
| 10 | Atopobium | 723 | 729 | 89 |
| 11 | Barnesiella | 200 | 129 | 56 |
| 12 | Bdellovibrio | 11 | 0 | 5 |
| 13 | Blautia | 40 | 11 | 14 |
| 14 | Buttiauxella | 0 | 22 | 11 |
| 15 | Butyrivibrio | 4718 | 6534 | 2522 |
| 16 | Chelatococcus | 66 | 187 | 59 |
| 17 | Clostridium_IV | 120 | 119 | 66 |
| 18 | Clostridium_XlVa | 91 | 35 | 30 |
| 19 | Clostridium_XlVb | 319 | 242 | 83 |
| 20 | Coprococcus | 78 | 13 | 27 |
| 21 | Desulfobulbus | 2721 | 2087 | 508 |
| 22 | Diplorickettsia | 79 | 0 | 22 |
| 23 | Ensifer | 106 | 303 | 78 |
| 24 | Enterorhabdus | 91 | 44 | 27 |
| 25 | Eubacterium | 441 | 242 | 66 |
| 26 | Fibrobacter | 812 | 780 | 155 |
| 27 | Hallella | 1205 | 2480 | 1021 |
| 28 | Howardella | 11111 | 8247 | 3245 |
| 29 | Lachnospiracea_IS | 143 | 76 | 27 |
| 30 | Microbacterium | 33 | 99 | 26 |
| 31 | Mogibacterium | 25 | 15 | 20 |
| 32 | Moryella | 0 | 41 | 14 |
| 33 | Nocardioides | 6 | 119 | 32 |
| 34 | Novosphingobium | 11 | 0 | 5 |
| 35 | Ochrobactrum | 1110 | 2330 | 712 |
| 36 | Oligosphaera | 31 | 39 | 35 |
| 37 | Olsenella | 126 | 16 | 38 |
| 38 | Opitutus | 19 | 0 | 10 |
| 39 | Oscillibacter | 47 | 127 | 43 |
| 40 | Paludibacter | 41 | 0 | 7 |
| 41 | Paraprevotella | 127 | 57 | 57 |
| 42 | Patulibacter | 16 | 0 | 8 |

| Item | Genus | MC | LC | MSE |
| --- | --- | --- | --- | --- |
| 43 | Phyllobacterium | 0 | 37 | 16 |
| 44 | Prevotella | 2266 | 1465 | 408 |
| 45 | Propionibacterium | 1252 | 1741 | 768 |
| 46 | Pseudochrobactrum | 51 | 89 | 40 |
| 47 | Pseudomonas | 18 | 23 | 12 |
| 48 | Pseudonocardia | 24 | 28 | 20 |
| 49 | Rhizobium | 282 | 304 | 131 |
| 50 | Rhodovulum | 14 | 0 | 7 |
| 51 | Roseburia | 27 | 0 | 13 |
| 52 | Ruminococcus | 322 | 191 | 28 |
| 53 | Saccharibacteria_genera_IS | 127 | 115 | 63 |
| 54 | Saccharofermentans | 36 | 45 | 21 |
| 55 | Schwartzia | 8 | 39 | 16 |
| 56 | Slackia | 3 | 77 | 23 |
| 57 | Solirubrobacter | 22 | 144 | 75 |
| 58 | Sphaerobacter | 169 | 471 | 194 |
| 59 | Sphaerochaeta | 8 | 26 | 11 |
| 60 | Sphingomonas | 89 | 205 | 50 |
| 61 | Spirosoma | 14 | 17 | 15 |
| 62 | Sporobacter | 20 | 32 | 14 |
| 63 | Staphylococcus | 255 | 486 | 215 |
| 64 | Streptococcus | 220 | 48 | 113 |
| 65 | Subdivision5_genera_IS | 161 | 297 | 88 |
| 66 | Succiniclasticum | 14 | 2 | 8 |
| 67 | Suttonella | 1553 | 1056 | 347 |
| 68 | Syntrophococcus | 6077 | 6170 | 536 |
| 69 | Thermus | 36 | 11 | 18 |
| 70 | Treponema | 418 | 248 | 97 |
| 71 | Vampirovibrio | 157 | 286 | 64 |
| 72 | Variovorax | 60 | 0 | 23 |
| 73 | Victivallis | 49 | 7 | 22 |
| 74 | C_Actinobacteria | 11 | 0 | 5 |
| 75 | C_Alphaproteobacteria | 273 | 443 | 113 |
| 76 | C_Betaproteobacteria | 0 | 24 | 12 |
| 77 | C_Clostridia | 3 | 56 | 16 |
| 78 | C_Deltaproteobacteria | 63 | 200 | 56 |
| 79 | F_Anaerolineaceae | 284 | 98 | 41 |
| 80 | F_Aurantimonadaceae | 29 | 22 | 16 |
| 81 | F_Clostridiales_IS_XIII | 272 | 162 | 157 |
| 82 | F_Coriobacteriaceae | 1248 | 1422 | 564 |
| 83 | F_Desulfovibrionaceae | 124 | 163 | 63 |
| 84 | F_Enterobacteriaceae | 4212 | 4000 | 540 |
| 85 | F_Erysipelotrichaceae | 179 | 121 | 64 |

| Item | Genus | MC | LC | MSE |
| --- | --- | --- | --- | --- |
| 86 | F_Lachnospiraceae | 7433 | 8222 | 419 |
| 87 | F_Neisseriaceae | 1241 | 985 | 425 |
| 88 | F_Phyllobacteriaceae | 34 | 89 | 31 |
| 89 | F_Porphyromonadaceae | 608 | 607 | 131 |
| 90 | F_Prevotellaceae | 885 | 836 | 112 |
| 91 | F_Rhodospirillaceae | 16 | 0 | 8 |
| 92 | F_Ruminococcaceae | 3210 | 3486 | 229 |
| 93 | F_Spirochaetaceae | 359 | 288 | 165 |
| 94 | F_Synergistaceae | 398 | 384 | 123 |
| 95 | F_Veillonellaceae | 292 | 174 | 79 |
| 96 | O_Bacteroidales | 2866 | 3486 | 727 |
| 97 | O_Campylobacterales | 132 | 94 | 17 |
| 98 | O_Chromatiales | 14 | 53 | 31 |
| 99 | O_Clostridiales | 15886 | 12450 | 3524 |
| 100 | O_Desulfuromonadales | 4 | 51 | 28 |
| 101 | O_Rhizobiales | 34 | 0 | 12 |
| 102 | O_Rhodospirillales | 0 | 216 | 108 |
| 103 | O_Selenomonadales | 103 | 185 | 59 |
| 104 | P_Bacteroidetes | 3438 | 3753 | 538 |
| 105 | P_Chloroflexi | 82 | 96 | 34 |
| 106 | P_Firmicutes | 1706 | 1802 | 576 |
| 107 | Unclassfied_Bacteria | 13606 | 14038 | 788 |

The consensus sequence of each Genus was annotated to the closest lineage blasting against RDA database.

F = family; O = order; C = class; P = phylum.

Genera were sorted based on their relative abundance in the rumen microbial community in a descending order.

# Table S3. Other possible functions of the first neighbors connected to the differentially expressed leptins on the network.

| **Gene** | **Neighbor** | **Regulation** | **Signaling Pathway** | **Function** |
| --- | --- | --- | --- | --- |
| CLEC16A | AP2A2 | Excretory system |  | Endocrine and other factor-regulated calcium  reabsorption |
|  |  | Nervous system |  | Synaptic vesicle cycle |
|  | ARHGAP35 | Cell motility |  | Focal adhesion |
|  |  | Cell motility |  | Regulation of actin cytoskeleton |
|  | CBLC | Endocrine system | Insulin signaling pathway | Inhibition of glucose uptake |
|  | DNAJC5 | Proteins |  | Heat shock proteins HSP40 / DNAJ |
|  | EFNA5 | Cytokines |  | Other growth factors |
|  |  | Development |  | Axon guidance |
|  | EFNB1 | Cytokines |  | Other growth factors |
|  |  | Development |  | Axon guidance |
|  | ETV6 | Development |  | Dorso-ventral axis formation |
|  | EWSR1 | Transcription |  | Spliceosome |
|  | EXTL3 | Glycan biosynthesis and metabolism |  | Glycosaminoglycan biosynthesis |
|  | FLOT2 | Endocrine system | Insulin signaling pathway | Inhibition of glucose uptake |
|  | GBGT1 | Glycan biosynthesis and metabolism |  | Glycosphingolipid biosynthesis - globo series |
|  | GOSR1* | Folding, sorting and degradation |  | SNARE interactions in vesicular transport |
|  | JUNB | Development |  | Osteoclast differentiation |
|  |  | Signal transduction | TNF signaling pathway | gene expression |
|  | KCNK2* | Ion channels |  | K+ channel, KCNK, K2px.x |

| **Gene** | **Neighbor** | **Regulation** | **Signaling Pathway** | **Function** |
| --- | --- | --- | --- | --- |
|  |  | Digestive system | Gastric acid secretion |  |
|  | LLGL2 | Cellular communication |  | Tight junction |
|  | LOC102173015 | Cell motility |  | Regulation of actin cytoskeleton |
|  | MAP3K4 | Endocrine system | GnRH signaling pathway | Gonadotropins gene expression & secretion |
|  | MCM5 | Replication and repair |  | DNA replication |
|  | MRI1 | Amino acid metabolism |  | Cysteine and methionine metabolism |
|  | MYH9 | Cellular communication |  | Tight junction |
|  | NOTCH3 | Development |  | Dorso-ventral axis formation |
|  |  | Signal transduction | Notch signaling pathway | gene expression |
|  | PTPRF | Signaling molecules and interaction |  | Cell adhesion molecules |
|  | PTPRJ | Cellular antigens |  | Proteins |
|  |  | Cellular communication |  | Adherens junction |
|  | SF3A1 | Transcription |  | Spliceosome |
|  | SIK2 | Endocrine system | Glucagon signaling  pathway | Glycolysis/glucogenesis |
| CLEC4E | ADORA2B | Metabolism of cofactors and vitamins |  | Porphyrin and chlorophyll metabolism |
|  | NFKBIA | Development |  | Osteoclast differentiation |
|  |  | Endocrine system | Adipocytokine signaling  pathway | Inhibition of glucose uptake |
|  |  | Signal transduction | cAMP signaling pathway | increased testicular (Anti-Müllerian Hormone)AMH  output (prepuberfal sertoli cell) |
| CLEC7A | CCL25 | Signaling molecules and interaction |  | Cytokine-cytokine receptor interaction |

| **Gene** | **Neighbor** | **Regulation** | **Signaling Pathway** | **Function** |
| --- | --- | --- | --- | --- |
|  | POLR3E | Transcription |  | RNA polymerase |
|  |  | Nucleotide metabolism |  | Purine metabolism |
|  |  | Nucleotide metabolism |  | Pyrimidine metabolism |
| LMAN2L | ATF6B | Aging | Longevity regulating  pathway - mammal | stem cell renewal, Hematopoietic regeneration |
|  |  | Circulatory system | Adrenergic signaling in  cardiomyocytes |  |
|  |  | Endocrine system |  | Aldosterone-regulated sodium reabsorption |
|  |  | Endocrine system |  | Insulin secretion |
|  |  | Endocrine system |  | Thyroid hormone synthesis |
|  |  | Nervous system |  | Dopaminergic synapse |
|  | CDC25A | Endocrine system |  | Progesterone-mediated oocyte maturation |
|  | CDC37 | Proteins | Glycosaminoglycan  binding proteins | Intracellular proteins |
